# Design and Characterization of a Novel p1025 Peptide-Loaded Liquid Crystalline System for the Treatment of Dental Caries

**DOI:** 10.3390/molecules21020158

**Published:** 2016-01-28

**Authors:** Giovana Maria Fioramonti Calixto, Matheus Henrique Garcia, Eduardo Maffud Cilli, Leila Aparecida Chiavacci, Marlus Chorilli

**Affiliations:** 1 Faculdade de Ciências Farmacêuticas, UNESP—Universidade Estadual Paulista, Campus Araraquara, Araraquara, SP 14800-850, Brazil; matheusgarcia3@hotmail.com; 2 Instituto de Química, UNESP—Universidade Estadual Paulista, Campus Araraquara, Araraquara, SP 14800-900, Brazil; eduardocilli@gmail.com

**Keywords:** nanostructured drug delivery systems, liquid crystalline system, bioadhesive polymers, tea tree oil, dental caries

## Abstract

Dental caries, mainly caused by the adhesion of *Streptococcus mutans* to pellicle-coated tooth surfaces, is an important public health problem worldwide. A synthetic peptide (p1025) corresponding to residues 1025–1044 of the adhesin can inhibit this binding. Peptides are particularly susceptible to the biological environment; therefore, a p1025 peptide-loaded liquid crystalline system (LCS) consisting of tea tree oil as the oil phase, polyoxypropylene-(5)-polyoxyethylene-(20)-cetyl alcohol as the surfactant, and water or 0.5% polycarbophil polymer dispersions as the aqueous phase was employed as a drug delivery platform. This system exhibited anticaries and bioadhesive properties and provided a protective environment to p1025 at the site of action, thereby modulating its action, prolonging its contact with the teeth, and decreasing the frequency of administration. LCSs were characterized by polarized light microscopy (PLM), small-angle X-ray scattering (SAXS), and rheological, texture, and bioadhesive tests. PLM and SAXS revealed the presence of hexagonal liquid crystalline phases and microemulsions. Rheological analyses demonstrated that the addition of polymer dispersions favored characteristics such as shear thinning and thixotropy, hence improving buccal application. Bioadhesion tests showed that polymer dispersions contributed to the adhesion onto the teeth. Taken together, LCS could provide a novel pharmaceutical nanotechnology platform for dental caries treatment.

## 1. Introduction

Dental caries is still a major public health problem, which can lead to the loss of teeth. Despite advances in dental treatment and prevention, such as restorative materials and oral hygiene campaigns, dental caries remain one of the most prevalent diseases worldwide [1].

The etiology of dental caries is mainly linked to factors and conditions that encourage the growth of bacteria, especially *Streptococcus mutans* (*S. mutans*). The first step of biofilm formation is the adhesion of *S. mutans* to pellicle-coated tooth surfaces [2].

Therefore, studies have evaluated several pharmacologically active molecules, such as peptides, to determine their potential to impede the growth or action of virulence factors, such as adhesins, that are related to the survival of *S. mutans* in the biofilm surface [3,4].

The antimicrobial peptide p1025 with the amino acid sequence Ac-QLKTADLPAGRDETTSFVLV-NH_2_, which is analogous to the 1025–1044 fragments of cellular adhesins of *S. mutans,* the bacterium responsible for the adhesion and biofilm formation on the oral mucosa, has demonstrated potential to combat dental caries. Kelly *et al*. reported that p1025 inhibited the binding of a cell surface adhesin of *S. mutans* to the salivary receptors *in vitro*, as confirmed by surface plasmon resonance [5]. Li *et al*. have demonstrated that a p1025-loaded dentifrice may selectively prevent *S. mutans* recolonization *in vivo* and *in vitro* [6].

However, major disadvantages of peptide administration are attributable to their physicochemical properties, including susceptibility to proteolytic degradation, short biological half-life, and high molecular weight, which decrease their bioavailability [7]. The incorporation of peptides into a clinically appropriate, safe, and effective delivery system for buccal administration represents an interesting option for the treatment of dental caries. 

Among the drug delivery systems, liquid crystalline systems (LCS) have attracted significant interest because they exhibit physical properties of both crystals and liquids. LCS can be obtained by mixing water, oil, and surfactant to afford different liquid crystalline mesophases: lamellar, hexagonal, and cubic [8,9,10]. 

It was of interest to employ tea tree oil (TTO) as the oil phase of a LCS, since TTO has been shown to be effective against *S. mutans* [11,12]. Furthermore, the incorporation of polyacrylic acid bioadhesive polymers, such as polycarbophil (PP), into the aqueous phase of a liquid crystalline system could also attenuate the adhesion at the action site. This is particularly important for buccal administration, since it is associated with several limitations mainly due to the continuous secretion of saliva (0.5 to 2 L/day), leading to dilution and possibly ingestion of the drug and ultimately, involuntary loss of the dosage form [13].

The aim of this study was to develop and characterize a nanotechnology platform for the treatment of dental caries. A novel p1025-loaded liquid crystalline system, comprising of polyoxypropylene-(5)-polyoxyethylene-(20)-cetyl alcohol (**PPCA**) as the surfactant, TTO as the oil phase and 0.5% PP dispersion or water as the aqueous phase was prepared and characterized by polarized light microscopy (PLM), small-angle X-ray scattering (SAXS), rheological, mechanical and bioadhesion assays.

## 2. Results and Discussion

**Figure 1:**
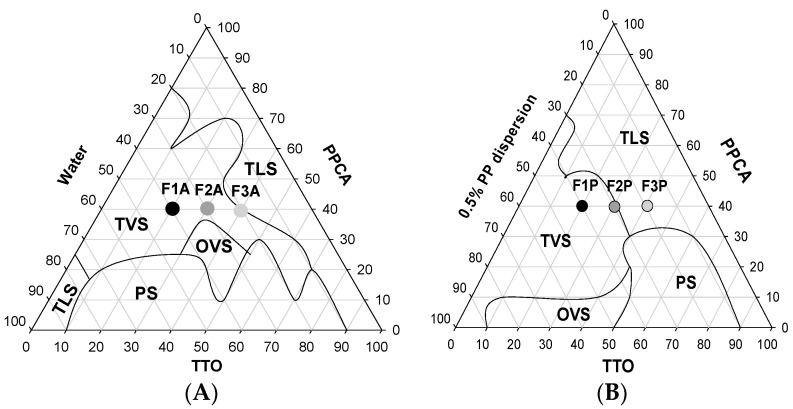
(**A**) Ternary phase diagram of polyoxypropylene-(5)-polyoxyethylene-(20)-cetyl alcohol (PPCA), tea tree oil (TTO), and water. F1-A, F2-A, and F3-A were the selected formulations for characterization; (**B**) Ternary phase diagram of PPCA, TTO, and 0.5% polycarbophil (PP) dispersion. F1-P, F2-P, and F3-P were the selected formulations for characterization. PS: Phase separation; OVS: opaque viscous systems; TLS: transparent liquid systems; and TVS: transparent viscous systems.

Figure 1A represents the pseudoternary phase diagram of the first investigated system: **PPCA** /TTO/water. A clear and transparent region was obtained in a wide region of the pseudo-ternary phase diagram and we observed a transition from transparent liquid system (TLS) to transparent viscous system (TVS), opaque viscous system (OVS), and phase separation (PS), with the proportion of the aqueous phase above 20%, TTO below 90%, and **PPCA** above 20%.

Figure 1B represents the pseudoternary phase diagram of the second investigated system: **PPCA** /TTO/0.5% PP. In this system, a larger transparent area and a lower region of phase separation than those of the first investigated system (Figure 1A) were obtained. Such behavior can be attributed to the presence of PP, which stabilizes the system. PP is a high molecular weight polymer that forms cross-links with divinyl glycol. It is insoluble in water but at neutral pH, the expansion of the chains when hydrated causes the formation of a strong gel due to neutralization of the carboxylic groups, and provides further stability to the system [14].

The viscosity of the systems increased at 40% surfactant in both systems with the addition of 20% to 40% water. This behavior is interesting because this dilution simulates, as closely as possible, the *in vivo* environment during buccal administration of the liquid formulations (water from the saliva), which results in a viscous formulation. We selected six formulations as highlighted in Figure 1A,B, which were F1-A, F2-A, and F3-A and F1-P, F2-P, and F3-P, respectively. The compositions of the formulations are shown in Table 1.

**Table 1:** The compositions (%) of LCS. TTO: tea tree oil, PPCA: polyoxypropylene-(5)-polyoxyethylene-(20)-cetyl alcohol, and 5% PP: 5% (*w*/*w*) polycarbophil dispersion.

Formulations	TTO (%)	PPCA (%)	Water (%)	5% PP (%)
F1-A	20	40	40	-
F2-A	30	40	30	-
F3-A	40	40	20	-
F1-P	20	40	30	10
F2-P	30	40	20	10
F3-P	40	40	10	10

**Figure 2:**
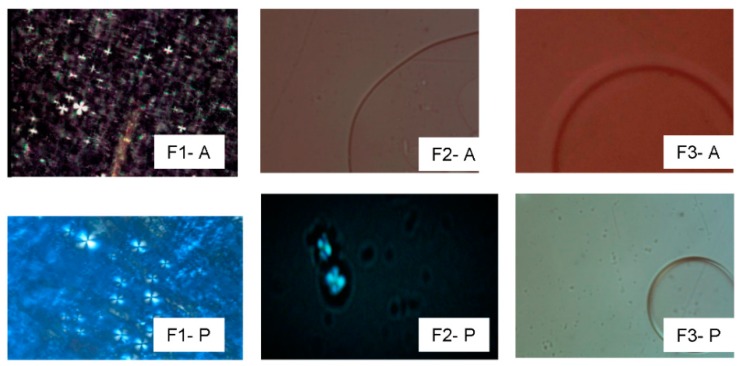
Polarized light microscopy photomicrographs of the formulations F1-A, F2-A, F3-A, F1-P, F2-P, and F3-P. Magnification 20×.

The photomicrographs of these formulations obtained by PLM are shown in Figure 2. F1-P, F2-P, and F1-A showed Maltese crosses, indicating the formation of a lamellar mesophase. F2-A, F3-A, and F3-P were represented by a dark field, which is a typical characteristic of microemulsions (MEs). MEs are clear, isotropic, and thermodynamically stable colloidal systems that are obtained spontaneously with surfactant-cosurfactant combinations [15]. The p1025-loaded formulations of F1-A, F2-A, F3-A, F1-P, F2-P, and F3-P showed the same patterns as the unloaded formulations.

SAXS tests were performed to confirm the arrangement of the liquid crystalline formulations obtained by PLM. This technique can detect the scattering of x-rays at angles *2*θ less than 10° related to the interplanar distances with nanometric dimensions; thus, it can characterize systems such as droplets, micelles, or crystalline structures by the average size and the distance between the scattering objects, even if they are not arranged in an orderly manner [15,16].

**Figure 3:**
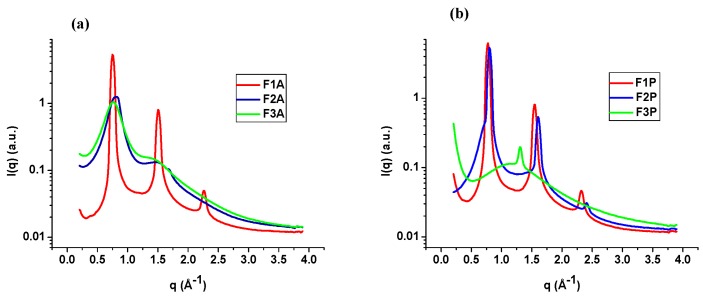
Small-angle X-ray scattering analysis of F1A, F2A, and F3A (**a**) and F1P, F2P, and F3P (**b**).

The intensity of the scattering patterns (I) *vs.* the scattering vector modulus q (Å^−1^) is shown in Figure 3.

Crystal liquid systems with random orientation can form one, two, and three-dimensional domain structures. The intensity I(q) produced by them displays a maximum form (or Bragg peaks) for specific values of the scattering vector q. The correlation distance (d) between the scattering objects can be calculated as d = 2π/qmax, where qmax is the q value at the peak of the intensity I(q) [16]. The ratio between the values of correlation distances can be useful to identify the liquid crystal arrangement.

F2A and F3A demonstrated liquid crystalline phase peaks but these peaks were broad, indicating the presence of a less ordered phase (probably a micellar solution) in admixture with an ordered liquid crystalline phase. The ratio between correlation distances listed in Table 2 for these samples is equal to 1:2, suggesting the presence of a lamellar arrangement. For sample F3P only a single well-defined peaks is observed, suggesting that this formulation is also a mixture of ordered and disordered phases. However, it is not possible to identify the coexisting liquid crystalline structure when only one peak is detected. Thus, further SAXS experiments are needed for understanding the effect of PPCA on the structure [17,18].

**Table 2:** Values of Q_max_ (A°) and the ration of the interplanar distances of the formulations.

Formulation	Q_max peak 1_	Q_max peak2_	Q_max peak3_	D-Spacing	d/d_h_
F1-A	0.75	1.50	2.26	8.37	1.00
F2-A	0.79	1.52	-	7.95	1.00
F3-A	0.76	1.46	-	8.26	-
F1-P	0.78	1.53	2.32	8.05	1.00
F2-P	0.80	1.60	2.41	7.85	1.00
F3-P	1.30	-	-	4.79	-

The relative position of the peaks of F1-A, F1-P, and F2-P follows the ratio 1:2:3 as shown in Table 2, indicating that these formulations had a higher organization, presenting a lamellar arrangement, which was also supported by the results of the PLM analysis (Malta crosses) [16].

Buccal formulations are subjected to various shear forces, including chewing, breathing, swallowing, and speech; therefore, it is important to evaluate the effects of such forces on the rheology of the formulations [19].

**Figure 4:**
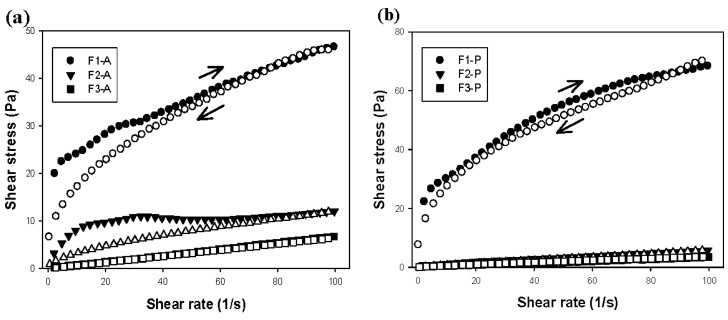
(**a**) Flow rheograms of the formulations F1-A, F2-A, and F3-A; (**b**) Flow rheograms of the formulations F1-P, F2-P, and F3-P. Closed symbol represents the upcurve and open symbol represents the downcurve. Standard deviations have been omitted for clarity; however, in all cases, the coefficient of variation of triplicate analyses was less than 10%. Data were collected at 37 ± 0.5 °C.

The relationship between shear rate (Pa) and shear stress (1/s) of the formulations is presented in Figure 4. From the data obtained by Equation (1), it was clear that F1-P, F2-P, F3-P, F1-A, and F2-A exhibited pseudoplastic behaviors (*n* < 1), whereas F3-A exhibited Newtonian behavior (*n* = 1).

Pseudoplasticity refers to a reduction in viscosity when the shear rate increases, which is a desirable feature of pharmaceutical formulations, since the reduction in viscosity facilitates the administration of the product [20,21,22,23]. 

Moreover, the incorporation of PP modified the hysteresis area of the systems. F1P showed a lower hysteresis area when compared to that of F1A. This phenomenon may be related to an increased stability of the system caused by the presence of PP, which reduced the structural disorganization caused by shear stress [14]. 

The presence of a hysteresis area indicates that the system exhibits time-dependent rheological behavior, *i.e.*, thixotropy. This behavior is present in materials that have the ability to recover its structure in the absence of stress, which is a desirable feature for bioadhesive buccal delivery. In the presence of shear forces (*i.e.*, the tongue) the system will flow and become less viscous; however, after a fixed time it can recover its structure and fixate more securely [19,20,21,22,23].

The rheology of all the systems showed desirable characteristics, as evidenced by the reduction in viscosity (shear-thinning), which facilitates product application, and the time-dependent recovery of the structure after administration allows for efficient bioadhesion.

**Figure 5:**
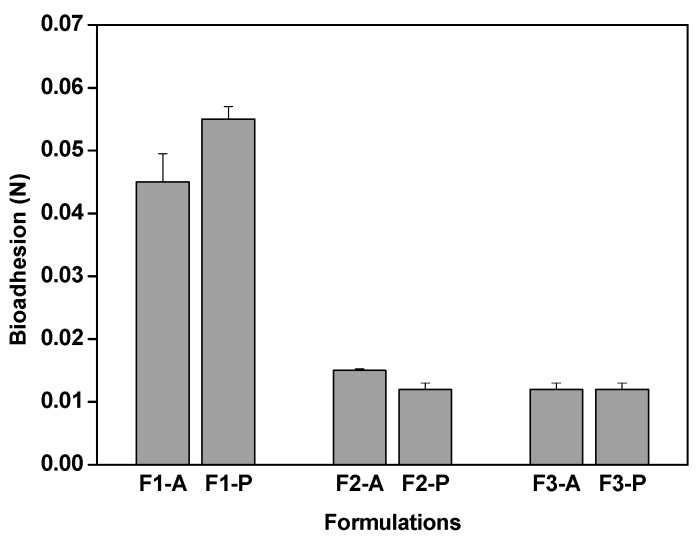
Bioadhesion of F1-A, F2-A, F3-A, F1-P, F2-P, and F3-P. Each value represents the mean (±SD) of at least seven replicates. Data were collected at 37 ± 0.5 °C.

The bioadhesion values of F2-A, F3 A, F2-P, and F3-P did not differ significantly from each other as shown in Figure 5, possibly because the amount of water in F2 and F3 was lower than that in F1, which contributed to the formation of a less viscous system and consequently, weaker bioadhesive strength [24,25].

However, the results demonstrated that F1-P had the greatest bioadhesion value, with similar values to that reported for hydrogel [14]. The addition of PP affected the rheology of the system, as evidenced by an increased viscosity, and improved the interaction between F1-P and the teeth [26].

There are several desirable texture attributes for formulations, which contribute to the clinical efficacy, patient acceptability, and ultimate approval of the product. These include efficient mechanical properties, such as ease of removal of the product from the container; product spreadability on the biological substrate such as the teeth, skin, or mucosa; good bioadhesion to ensure retention at the site of application, and viscosity that allows efficient releasing and absorbing of the drug [14,22,27]. These properties will affect the applicability of the formulation at the administration site and consequently the outcome of therapy [25].

From the force-time plot of the TAXT program, the following mechanical parameters can be described: (i) hardness (force required to achieve a given strain); (ii) adhesiveness (an amount which simulates the necessary work to overcome the forces of attraction between the sample surface and the probe surface that the sample comes into contact with); (iii) compressibility (force per unit of time required to deform the product during the first compression cycle of the probe); and (iv) cohesion (the ratio between the area of positive force in the second compression to the first compression) [14,22,26,28].

The formulations identified as liquid systems (F2-A, F3-A, F2-P, and F3-P) were not subjected to mechanical property analysis including hardness, compressibility, cohesiveness, and adhesiveness tests, because they did not display mechanical resistance to flow [22].

The mechanical parameters of F1-A and F1-P are shown in Table 3. Statistical analysis indicated that F1-A and F1-P did not differ significantly, suggesting that the presence of polymers did not alter the mechanical properties of the formulations, such as compressibility, hardness, adhesiveness, and cohesiveness.

**Table 3:** Mechanical properties (hardness, compressibility, adhesiveness, and cohesiveness) of F1A and F1P. Each value represents the mean ± standard deviation (*n* = 7).

Formulations	Hardness (N)	Compressibility (N.s)	Adhesiveness (N.s)	Cohesiveness
F1-A	0.031 ± 0.002	0.398 ± 0.01	0.152 ± 0.02	0.860 ± 0.04
F1-P	0.022 ± 0.003	0.297 ± 0.03	0.299 ± 0.01	± 0.05

The texture profile analysis (TPA) values were consistent with the findings of studies that assessed systems containing polyoxypropylene-(5)-polyoxyethylene-(20)-cetyl alcohol as the surfactant, oleic acid or mineral oil as the oil phase, and water as a platform for the development of topical drug delivery systems [29].

Therefore, the developed formulations should be further investigated since they demonstrated promising properties as a drug delivery platform for the treatment of buccal diseases such as dental caries.

## 3. Experimental Section

### 3.1. Materials

Polyoxypropylene-(5)-polyoxyethylene-(20)-cetyl alcohol (PPCA) was purchased from Croda (Campinas, Brazil). Polycarbophil (Noveon^®^ AA-1) was from Lubrizol (Cleveland, OH, USA), tea tree oil (TTO) was from Sigma-Aldrich (Steinheim, Germany) and triethanolamine was obtained from Synth^®^ (Diadema, Brazil). High-purity water was prepared by using a Millipore Milli-Q Plus purification system (Bedford, MA, USA).

### 3.2. Methods

#### 3.2.1. Synthesis, Purification, and Preparation of the Peptide

The peptide p1025 with sequence Ac-QLKTADLPAGRDETTSFVLV-NH_2_ was synthesized and purified according to a recently reported method [10]. The peptide was solubilized in sterile deionized water with 0.1% acetic acid (CH_3_COOH) at a concentration of 1000 μg·mL^−1^ prior to use and stored in a freezer at −20 °C until use. The molecular weight of the peptide p1025 is 2202.5 g/mol.

#### 3.2.2. Construction of the Pseudoternary Phase Diagram

To determine the concentration range of the components that form lyotropic liquid crystals, two pseudoternary phase diagrams were constructed at 25 ± 0.5 °C. The first diagram was constructed by weighing and mixing PPCA, TTO, and water. The second was constructed by weighing different combinations of PPCA, TTO, and 0.5% (*w*/*w*) PP dispersion. PP polymer was dispersed in water at 5% (*w*/*w*) and homogenized at 2000 rpm in a mechanical stirrer for approximately 10 min. The pH of the PP dispersion was adjusted to 6.0 with triethanolamine and manual agitation [14]. After, 5% PP dispersion was added to each formulation (as shown in Figure 1B) to obtain a final PP concentration of 0.5%. All samples were maintained at 25 ± 0.5 °C for 24 h to reach equilibrium. Each final formulation had a pH of 5.5. Each phase was identified by PLM, as described below. Based on the structures identified, the ternary phase diagrams of the systems were constructed with Sigma Plot Software (Figure 1).

#### 3.2.3. Polarized Light Microscope

A small amount of the formulation was placed on a glass slide and covered with a coverslip; it was then analyzed using a polarized light microscope (Jenamed, Carl Zeiss, Oberkochen, Germany). The homogeneity of the dispersion and signs of anisotropy or isotropy were examined at 20× magnification at room temperature (25 ± 0.5 °C).

#### 3.2.4. Small-Angle X-ray Scattering 

The structural arrangements of the LCSs were analyzed by SAXS using the Brazilian Synchrotron Light Laboratory instrument (LNLS, Campinas, Brazil) equipped with a type Si (111) monochromator with a wavelength (λ) of 1.608 Å that yields a horizontally focused beam. A vertical Pilatus 300K SAXS detector located at 858.45 mm from the sample and a multichannel analyzer 13 were employed to record the intensity of the scattering I(q) from 0.1 to 3.8 Å^−1^ at 25 °C. The scattering particles in the system without sample were subtracted from the total intensity of the sample, as a function of the modulus of the scattering vector *q*, q = 4π sinθ/λ, where λ is the wavelength and *2*θ is the scattering angle. The intensities of all samples were measured in relative units, but a quantitative comparison of the measurements was standardized under the same experimental conditions.

#### 3.2.5. Rheological Measurements

The flow properties of all the systems were analyzed in triplicate by using a controlled stress rheometer AR2000 (TA Instruments, New Castle, DE, USA) equipped with parallel plate geometry (diameter of 35 mm diameter and a sample gap of 0.020 mm), at 37 ± 0.1 °C. The test was performed using a controlled shear rate procedure ranging from 0.01 to 100 s^−1^ and back, each stage lasting for 120 s with an interval of 10 s between the curves. The consistency index and flow index were determined from the power law described in Equation (1) to quantify the flow behavior:

(1) $$\tau =k\cdot \gamma^{\eta }$$

where “τ” is the shear stress; “γ” is the shear rate; “*k*” is the consistency index and “η” is the flow index [14].

#### 3.2.6. Texture Profile Analysis 

TPA was performed using a TA-XTplus texture analyzer (Stable Micro Systems, Surrey, UK) in the TPA mode. The formulations (8 g of each sample) were centrifuged at 4000 rpm for 10 min (5810R, Eppendorf, New York, NY, USA) and the cylindrical analytical probe (1 mm diameter) was lowered (1 mm·s^−1^) until it reached the sample. Hydrogels were compressed twice (0.5 mm·s^−1^; 10 mm depth; 5 s delay period). Hardness, compressibility, adhesiveness, and cohesion were calculated from the force-time curves through the program Expert Texture Exponent. Seven replicates were analyzed at 25 ± 0.5 °C.

#### 3.2.7. *In Vitro* Bioadhesion Strength

##### Saliva Collection

Saliva was collected from 15 healthy donors with informed consent and ethical approval from the Committee of Ethics of Research of the School of Pharmaceutical Sciences of Araraquara, Brazil, under protocol 15/2011. The donors did not use any antibiotics, mouthwashes, or other medication known to affect the composition and salivary flow rate in the last 3 months. Saliva was collected in ice-cooled conical centrifuge tubes of 50 mL volume. Next, an equal amount of saliva from each donor was mixed, homogenized, and centrifuged at 10,000 *g* for 5 min at 4 °C [12]. Then, the saliva was filtered using a filtration system with a membrane of 0.22 µm [13]. The saliva that was not used immediately was stored at −70 °C. 

##### Preparation of Discs

Sound bovine permanent central incisors were collected and scaled for the removal of periodontal tissue remnants and other debris. Teeth with enamel cracks, hypoplasia, and calculus in the middle third of the root or other morphological alterations were excluded. Enamel blocks were cut transversally from the middle third of the buccal surface of each tooth with a water-cooled, double-faced diamond disc (KG Sorensen, Barueri, SP, Brazil). Next, the specimens were rounded with a high-speed, water-cooled cylindrical diamond bur (1095; KG Sorensen) to obtain specimens of 1 cm diameter containing enamel. Teeth surfaces were polished with wet 200-grit silicon carbide paper (T469-SF- Norton; Saint-Gobain Abrasivos Ltda., Jundiaí, SP, Brazil) to regularize the surface [14].

##### Bioadhesion Measurement by the Tensile Strength Method

A TA-XTplus texture analyzer (Stable Micro Systems) was used to measure the tensile strength. The teeth model was fastened to the upper movable probe as described above, and the formulation sample was located on the lower platform. The upper probe was lowered until it was in contact with the sample and was kept in contact for 60 s without any force applied. After, the probe was raised at 0.5 mm/s, and the force required for detachment was registered. The tensile work (g·s), which is proportional to the area under the force–time curve, was used to describe the bioadhesive characteristics. Seven replicates were analyzed at 37 ± 0.5 °C.

## 4. Conclusions

Polarized light microscopy and small-angle X-ray scattering analysis was employed to characterize a LCS comprising of tea tree oil, polyoxypropylene-(5)-polyoxyethylene-(20)-cetyl alcohol, and 5% polycarbophil polymer dispersions as the aqueous phase. Rheological, mechanical, and bioadhesion tests demonstrated that the presence of polymer dispersions improved the interaction of LCS with the teeth surface. Taken together, our findings suggest that this novel LCS is a potential platform for the treatment of caries.
